# Institutional Notes and News

**Published:** 1910-12-03

**Authors:** 


### Institutional Notes and News.

Mr. John Burns was busy over institutional matters again last Saturday, November 26, when he opened the new infirmary for the Wandsworth Union. The importance of the occasion will be realised when we add that £90,000 has been spent on building and equipment, and that the new infirmary will contain 600 beds. The growing importance every year of the Poor-law infirmaries and the necessity for the maintenance in them of nothing less than the highest standard have been recognised consistently by Mr. Burns, whose good work on behalf of Poor-law patients it would be hard to exaggerate. We take the following points from his speech : " There is a determination on the part of the Local Government Board, in combination with local administrative authorities, to put the Poor-law medical service on a sound footing, and to staff and equip it in a manner equal to the staffing and equipment of the best general and special hospitals. While pauperism has greatly decreased, there has been an increase in its cost. At present we have the lowest outdoor pauperism ever recorded for England and Wales, and the indoor pauperism of the metropolis was now the lowest for three years. Indoor pauperism has remained stationary during the past ten years, because the workhouse was no longer the Bastille it used to be, and because children within the wards of the poorhouse no longer experienced the treatment that was meted out to the Oliver Twists of fifty years ago. Over 50 per cent. of the indoor poor were now in special institutions, where their ailments were better treated than in the old mixed general workhouse, which was a combination of the lunatic asylum and the gaol, with a little bit of street-corner thrown in. If we had the old standard of relief and sternness of administration the figures of pauperism would be lower than they were, but the increased cost is due entirely to the better treatment of the aged, the sick, and the children. It is better economy to spend £50 in getting rid of sickness before the patient is fourteen than to spend £300 in palliating his symptoms after he has turned forty. There were 200,000 inmates in the London infirmaries, and over a million a year was spent on them. Public bodies, therefore, could hardly concern themselves with a matter of greater importance to the community, and I am glad that Parliament recognises this, and supports me most generously and consistently. While the Utopians have been agitating, the Wandsworth Guardians have been quietly doing." The same praise might be applied with equal aptness to the President of the Local Government Board.

**Mr. Burns at Wandsworth Infirmary.**

Ancoats Hospital, Manchester, has to record a surprising increase in the number of accidents treated by it during the past twelve months, which increase was owing, according to Lord Rotherham, to its position in the busiest part of the city. Taking the figures in the report, we find that while there was an increase of 5,810 in the number of patients treated, 5,194 more accident cases have been received. Statistics are proverbially difficult to analyse, but it would be interesting to know the contributory causes to this. The income was, roughly, £1,000 less than the expenditure, which leaves the hospital with a debt of £7,560 to the bank. The Board of Management is appealing for £20,000 in donations to put the institution upon a sound financial basis, also for an increased subscription list of £1,500. In response £7,072 has been received, also £620 in new subscriptions. The great increase of the number of patients necessitates the constant and complete occupation of the beds, in consequence of which many patients are treated as out-patients who ought to be admitted to the wards, others have to wait for admission so long that their chances of recovery are jeopardised. Though it is not the intention of the Board to increase the number of beds at the present time, there can be no doubt that two extra wards are most urgently needed, but this cannot be done owing to the lack of funds. The hospital being situated in the poorest parts of the City, where there are no rich or middle class living, is not supported as it should be, and no doubt it is due to the fact of not being brought before the public eye. The Lord Mayor of Manchester, Mr. Charles Behrens, referred to the number of accidents and the hospital's situation, remarking that the two were interdependent and should appeal most strongly to the giving public. " You are too modest," he said, " and do not advertise enough, or employ such sensational methods as other institutions." It must have occurred to the many who laughed at this remark that in it lay perhaps the germ of good propaganda, and that " the hospital which does not advertise " might become a very good advertisement indeed ! Owing to the removal of the Royal Infirmary much more work has been put upon this institution; in fact, at the present time Ancoats Hospital is the chief hospital in Manchester for accident cases. Last year the Ancoats Hospital treated 23,279 casualties, the largest number recorded by any hospital in the City of Manchester—in fact, it is safe to say, by any hospital in the North of England. The total number of patients treated, inclusive of casualties, in-patients and home-patients, was 36,835.

**Ancoats Hospital, Manchester.**

Owing to the isolated position of Frickley Colliery—it is some distance away from Doncaster and Barnsley Hospitals—the owners (Carlton Main Colliery Company) have arranged to build a hospital for the benefit of the men employed under them at Frickley. It is stated that a contract has been secured by Messrs. Gill and Son, of Doncaster, for its erection. The site is a piece of land about two acres in size adjoining the road from South Emsall to the colliery, and has generously been presented by Mr. W. W. Warde Aldam, of Frickley Hall. The hospital and other buildings will be erected in what is known as the cottage style of red brick and stucco, with red-tiled roofs, and the cost will be about £5,000. The buildings have been designed by Mr. E. H. Walker, of Doncaster, and will include two main wards. The male ward will contain ten beds and that for females five, in addition to which there will be two small private wards for paying patients. The total accommodation of the hospital, which it is intended should be used primarily for accident cases from the colliery, may be increased to twenty beds. The promoters of the scheme, the Carlton Main and Grimethorpe Collieries Company, have been loyal supporters of the Beckett Hospital, Barnsley, for many years.

**A New Colliery Hospital.**

The annual meeting, held last week, of the subscribers to the Western Infirmary, Glasgow, shows that the hospital and its supporters are busy with plans to meet the demands on that growing institution. Sir Mathew Arthur, chairman of the managers, who presided, announced that by the generosity of an anonymous friend £3,500 had been handed to the managers for an operation theatre on the first floor. The new wing, which will have accommodation for almost 100 beds, is expected to be ready for opening early in 1911. The other

**Western Infirmary, Glasgow**

extension contemplated is an administration block with an admission department. The new theatre, which was hoped for also, has been given, as noted above. The clinical laboratory, for which £5,000 was received as a gift, will be ready for occupation by the end of the year. Thus the constructive side of the past year's work has been extremely satisfactory, but a variety of causes has led to a deficit on the hospital's expenditure. An increase has been shown in almost every class of subscriptions, but the net increase of £1,144 was £336 less than the increase in the ordinary expenditure, an increase due to the greater number of patients treated. Each special department had received more cases, and the out-patients had grown also in numbers. The total ordinary income was £22,949, and the ordinary expenditure was £37,972, the deficit of £15,024 having been met by transferring the balance of their extraordinary income to the maintenance account and by taking £1,000 from their stocks. Sir William Bilsland remarked that there was an average of 400 persons waiting admission.

**Worcester General Infirmary.**

A special meeting of the governors of the Worcester General Infirmary was held last week to discuss finance. The Chairman explained that during the past ten years the ordinary income had averaged £4,700, and the ordinary expenditure £7,500, the excess of expenditure over income during those years amounting to £28,000. Legacies amounting to £20,000 had been received and treated as income, and £8,500 had been taken from the funded property of the institution. The invested property now amounted to something less than £50,000, of which £30,000 was ear-marked. The position was serious, and he wished there had been a larger gathering of governors to consider it. The Rev. C. Jerram Hunt said they were placed in a dilemma; either they had to lessen the beneficial output of the institution, or to go on spending money to make the infirmary efficient. He moved that the infirmary should be maintained on its present lines, no expense being spared to make and keep it efficient. Canon Longhurst did not agree, and said that he saw no prospect of any substantial increase in the subscriptions, and moved this amendment: "That the Executive Committee be requested to prepare a scheme whereby the expenditure of the Worcester Infirmary be made to approach more nearly the level of its income, and submit a scheme before a meeting of the governors to be called on the third Monday in January 1911," which, however, was defeated. The Chairman said that the Committee had done its best to reduce expenditure. This was not a business undertaking, and, though he might be wrong from a financial point of view, how could it be said this was a charitable institution if it turned people from its doors? They should do what they could to economise, but not cripple the work of the infirmary. Mr. Longhurst's motion, "That an annexe be built to provide for the more efficient treatment of children and out-patients, and the proposals of the Worcester City King Edward VII. Memorial Committee to apply its fund for the building of that annexe be gratefully accepted," was carried.

